# Anastrozole-related acute hepatitis with autoimmune features: a case report

**DOI:** 10.1186/1471-230X-11-32

**Published:** 2011-03-31

**Authors:** Alessandro Inno, Michele Basso, Fabio M Vecchio, Valentina A Marsico, Eleonora Cerchiaro, Ettore D'Argento, Cinzia Bagalà, Carlo Barone

**Affiliations:** 1 Division of Medical Oncology, Catholic University of the Sacred Heart, Rome, Italy; 2 Institute of Pathological Anatomy, Catholic University of the Sacred Heart, Rome, Italy

**Keywords:** Anastrozole, drug-induced liver injury, autoimmune hepatitis, hepatotoxicity, antinuclear antibodies, case report

## Abstract

**Background:**

Two cases of acute hepatitis occurring during treatment with anastrozole have previously been reported, but the underlying mechanisms of liver injury are still uncertain. We report the case of anastrozole-related acute hepatitis with some autoimmune features.

**Case presentation:**

A 70-year-old woman developed acute hepatitis associated with serum antinuclear antibodies during anastrozole treatment; after drug withdrawal, liver function parameters rapidly improved and serum auto-antibodies were no longer detectable.

**Conclusions:**

Anastrozole-induced hepatotoxicity is a very rare event. Drug-drug interactions or metabolically-mediated damage might be involved, with a possible role of individual susceptibility. Our report suggests that an immune-mediated mechanism may also be considered in anastrozole-related liver injury.

## Background

Anastrozole is a selective aromatase inhibitor approved for the treatment of postmenopausal hormone-sensitive breast cancer [1]. The principal side effects are osteoporosis resulting in increased incidence of bone fractures, hypercholesterolemia with no significant increase in cardiovascular risk, and musculoskeletal events such as arthralgia and myalgia [2].

Other adverse events have been less frequently reported, including two cases of severe acute hepatitis [3,4]; however, the underlying mechanisms of anastrozole-related liver injury are still poorly understood. We report a case of anastrozole-related acute hepatitis associated with serum antinuclear antibodies, suggesting a possible role of the immune system in anastrozole-related liver damage.

## Case presentation

A 70-year-old woman underwent left upper-lateral quadrantectomy and axillary dissection for a high grade 2,5 cm intra-ductal breast carcinoma in June, 2008. Tumor cells were ER positive and PR negative, immunohistochemistry staining for c-ErbB2 was negative, Ki67 was 35%; no metastases were found in 21 axillary lymph nodes. Chest radiography, abdominal ultrasound and bone scintigraphy showed no distant metastases. Anti-estrogen treatment with anastrozole (1 mg/day) was started in August 2008, and whole breast radiotherapy (5000 cGy) was delivered from November to December 2008, followed by a boost to the tumor bed (1000 cGy). The only concomitant complaint was arterial hypertension, which had been treated with ramipril, bisoprolol and manidipine for about three years prior to the onset of breast cancer. Before starting anastrozole, liver function tests were normal: aspartate aminotransferase 18 U/l (normal range: 7-45), alanine aminotransferase 25 UI/l (normal range: 7-45), alkaline phosphatase 158 U/l (normal range: 98-279), total bilirubin 1.2 mg/dl (normal range:0.3-1.2) and gamma-glutamyltransferase 10 (normal range 5-36).

**Figure 1:**
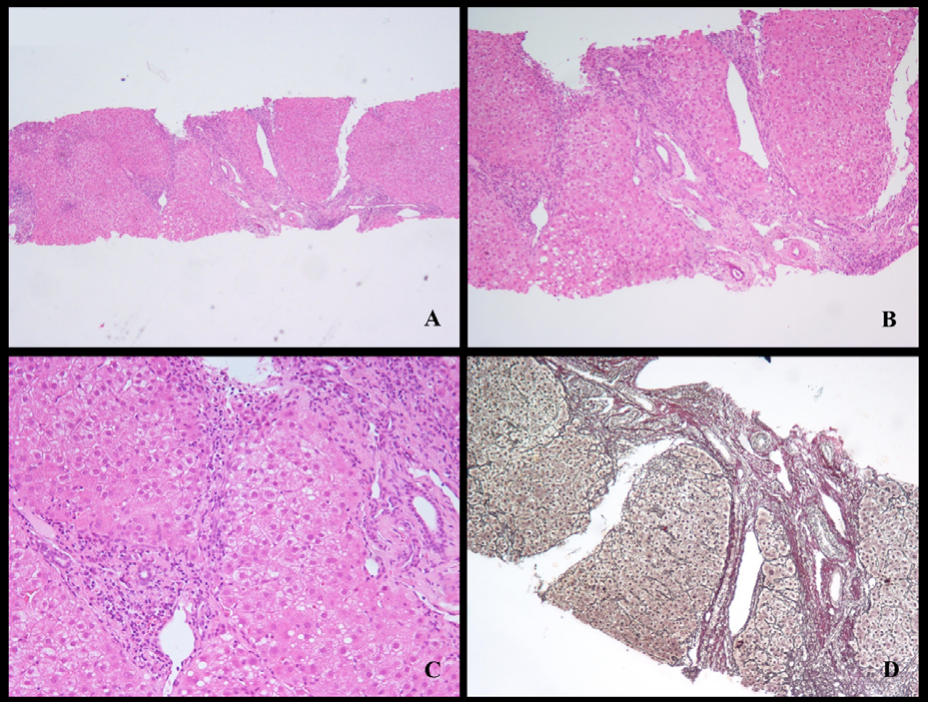
**Photomicrographs of liver biopsy, with hematoxylin/eosin (A, B, C) and reticulin stain (D), revealing a pattern of mild steatosis, moderate inflammatory changes and moderate to severe fibrosis (Ishak score = 5-6)**.

In January 2009, i.e. four months after beginning anastrozole, the patient developed mild asthenia. Laboratory tests showed changes consistent with a mixed hepatocellular and cholestatic liver injury: aspartate aminotransferase 640 U/l, alanine aminotransferase 1344 U/l, alkaline phosphatase 355 U/l, total bilirubin 3.54 mg/dl, conjugated bilirubin 2.29 mg/dl, gamma glutamyltransferase 234 U/l. Anastrozole was withdrawn and the patient was hospitalized. Hepatitis A, B and C and Epstein-Barr virus serology were negative; IgM-type antibodies (Abs) against cytomegalovirus were also absent. Anti-smooth muscle, anti-liver and kidney microsomal, anti-neutrophil cytoplasmic, anti-mitochondral, anti-native DNA, anti-extractable nuclear antigens and anti-gastric parietal cell Abs were negative. Anti-nuclear Abs, however, were positive with 1:80 titer and a speckled pattern. Abdominal ultrasound and CT scan showed no hepatic lesions, only a mild dilatation of the intrahepatic biliary tract without biliary stones or evidence of other possible causes of biliary duct obstruction. A liver biopsy was performed and histological examination revealed a pattern of mild steatosis (10%), with moderate inflammatory activity and moderate to severe fibrosis, totalizing a 5-6 score according to Ishak's classification (Figure 1) [5].

After anastrozole discontinuation, a dramatic clinical and laboratory improvement was observed. Mainly for this reason other imaging procedures were considered unncecessary. Liver parameters returned to normal ranges in one month; subsequently, the anti-estrogen schedule was switched to tamoxifen with no side effects. Interestingly, twelve months later even serum anti-nuclear Abs were undetectable.

## Discussion

In clinical trials, anastrozole has generally shown a good liver safety profile [6]. Our patient, however, developed acute liver damage during treatment with this aromatase inhibitor, but achieved a prompt and full recovery after discontinuation of the drug. Overall, the time lapse between drug exposure and the onset of hepatitis, the age of the patient, the exclusion of other non-drug-related causes, the improvement achieved after drug withdrawal and other reported cases of anastrozole-related hepatitis, allow our case to achieve a score of 7 according the Roussel-Uclaf Causality Method; this result suggests a probable correlation between anastrozole and liver damage [7].

Only two other cases of hepatitis occurring during treatment with anastrozole have previously been reported. The two patients, aged 58 and 89 years, developed acute, mainly cholestatic liver damage during anastrozole therapy; in both patients liver function tests improved after drug withdrawal [3,4]. After excluding other etiologic factors, anastrozole was considered the most probable cause of liver injury. However, in the case reported by Zapata *et al*, liver biopsy was not performed, preventing any reliable pathogenetic assumption [3]. In the case reported by de la Cruz *et al*, liver biopsy revealed diffuse liver cell necrosis in acinar zone 3, the preferred location of most drug-metabolizing P450 isoenzymes, but no inflammatory changes [4]. The authors considered these findings compatible with a metabolically-mediated hepatocellular liver injury. Indeed, anastrozole is extensively metabolized in the liver by N-dealkylation, hydroxylation and glucuronidation; thus, a genetic polymorphism of any enzyme involved in drug detoxification could cause an accumulation of the parental drug or its metabolites, predisposing to anastrozole-induced liver toxicity. Moreover, a cytochrome-mediated drug-drug interaction might modify plasma anastrozole concentrations, increasing the risk of toxic effects. However, both the potential and the mechanisms of anastrozole hepatotoxicity remain uncertain.

In the two previously reported cases, autoantibodies were not found, whereas in our patient serum antinuclear Abs were detected when hepatitis was recognized. Interestingly, Abs were cleared after anastrozole withdrawal, suggesting a link between antinuclear Abs and exposure to the drug. The absence of a baseline value only minimally affects this conclusion, assuming that the normal laboratory condition reached after anastrozole withdrawal would be similar to the normal baseline condition.

A metabolic interaction of anastrozole with simultaneously administered bisoprolol and manidipine seems unlikely in our patient. Bisoprolol is metabolized by CYP3A4, and manidipine can inhibit CYP2C9, both cytochromes being in turn down-regulated by anastrazole [8]. Although a cytochrome-mediated interaction could not be excluded, there are no pharmacological studies that show a significant risk of CYP-mediated drug-to-drug interactions with anastrozole, nor do bisoprolol or manidipine seem to have a significant potential for liver injury [9,10].

In our patient, liver biopsy showed no histological features typical of autoimmune hepatitis (such as bridging necrosis, hepatic rosettes or clusters of plasma cells extending from the portal tract into the parenchyma); nor, however, did it exhibit a pattern specific to any other pathogenesis. Unfortunately, a complete score according to Hennes' diagnostic criteria for autoimmune hepatitis could not be calculated, as serum immunoglobulin levels were not determined at the onset of hepatitis [11]. However, the temporal relationship with anastrozole, the presence of antinuclear antibodies and the absence of viral hepatitis or other hepatic diseases, taken together, are suggestive of an autoimmune mechanism in the pathogenesis of this drug-induced hepatitis. The rapid improvement of liver function together with the disappearance of antinuclear Abs after withdrawal of the aromatase inhibitor suggests that anastrozole may have induced hepatitis with autoimmune features rather than brought to light an underlying liver disease. Even though a long-term follow-up was not available for our patient, anastrozole-induced hepatitis seems a reversible, positive-prognosis and self-recovering toxic event. These characteristics are similar to those of autoimmune hepatitis induced by nitrofurantoine or minocycline, the two drugs mainly involved in immune-mediated liver injury [12].

Interestingly, the use of anastrozole is associated with a higher incidence of arthralgias and inflammatory arthropathies than either placebo or tamoxifen, and Laroche *et al *found antinuclear antibodies (titer > 1:160) in nine of twenty-four women treated with aromatase inhibitors who developed arthralgias [13]. Autoimmune diseases such as Sjogren's syndrome, Schonlein-Henoch purpura and Rheumatoid Arthritis have also been reported in patients treated with anastrozole [14,15]. Taken together, these reports support the hypothesis that anastrozole might affect immune regulation, probably by reducing self-tolerance, but the mechanism of this possible interference is unknown.

## Conclusions

Anastrozole-related hepatotoxicity is a rare and perhaps underestimated event whose pathogenesis is not clear; several factors, including an immune-mediated mechanism, could be involved. In the present case and in the others so far reported, a complete recovery followed anastrozole withdrawal. However, when anastrozole-related liver injury is suspected, an exhaustive study is necessary, including liver biopsy and a careful immunologic assessment in order better to understand the mechanisms of toxicity.

## Consent

Written informed consent was obtained from the patient for publication of this case report and any accompanying images. A copy of the written consent is available for review by the Editor-in-Chief of this journal.

## Competing interests

The authors declare that they have no competing interests.

## Authors' contributions

All the authors significantly contributed to the management of the patient. In particular, FMV performed the histological evaluation. AI collected the data and wrote the report, and was involved in drafting the manuscript. CaB revised the manuscript critically for important intellectual content. All the authors read and approved the final manuscript.

## Pre-publication history

The pre-publication history for this paper can be accessed here:

http://www.biomedcentral.com/1471-230X/11/32/prepub
